# The guiding significance of calcaneal tuberosity integrity in the surgical treatment of calcaneal fractures: a retrospective case–control study

**DOI:** 10.1007/s00264-026-06927-8

**Published:** 2026-06-23

**Authors:** Gang Luo, Xing Du, Zenghui Wang, Weidong Ni

**Affiliations:** Department of Orthopaedic Surgery, The First Affiliated Hospital of Chongqing Medical University, Chongqing, China

**Keywords:** Calcaneal fracture, Tuberosity integrity, Ligamentotaxis, Minimally invasive surgery, Surgical approach, Sanders classification, Operative time

## Abstract

**Background:**

The optimal surgical approach for displaced intra-articular calcaneal fractures remains contentious. While minimally invasive surgery (MIS) reduces soft-tissue complications, its application in complex fractures is debated. Ligamentotaxis, a cornerstone of MIS, requires an intact calcaneal tuberosity as a fulcrum. This study evaluates the utility of preoperative tuberosity integrity assessment in guiding surgical approach selection.

**Methods:**

In this retrospective case–control study, 118 patients (2019–2022) were allocated to two treatment algorithms due to an institutional protocol evolution. Group A (Sanders-guided, n = 51) received MIS for Sanders type II/III and ELA for type IV. Group B (tuberosity-guided, n = 67) received MIS if the tuberosity was intact or an extended lateral approach (ELA) if comminuted. Outcomes included operative metrics, radiographic reduction (Böhler’s angle, facet step-off), functional scores (AOFAS, Maryland Foot Score), VAS pain scores, and complications.

**Results:**

No significant differences were observed in radiographic or functional outcomes between the two groups. However, the tuberosity-guided approach suggested a potential reduction in operative time for fractures with comminuted tuberosity (93.5 ± 6.1 min vs. 103.2 ± 4.7 min, p = 0.017, a preliminary finding due to the small sample size). Analysis of the entire cohort (n = 118) revealed that the wound complication rate was notably higher when comminuted tuberosity fractures were treated with STA (2/2, 100%) compared to ELA (1/9, 11.1%, p = 0.011). Furthermore, among all STA cases, patients with comminuted tuberosity had a markedly higher observed complication rate than those with intact tuberosity (100% vs. 9.5%, *p* < 0.001).

**Conclusion:**

Preoperative assessment of calcaneal tuberosity integrity offers a potentially valuable adjunct to the Sanders classification for selecting the surgical approach in displaced intra-articular calcaneal fractures. It achieves comparable clinical outcomes. Given the exploratory nature of subgroup analyses based on a limited number of comminuted fractures, the findings regarding enhanced operative efficiency and complication profiles are preliminary and require validation in larger, prospective cohorts.

**Supplementary Information:**

The online version contains supplementary material available at 10.1007/s00264-026-06927-8.

## Introduction

Displaced intra-articular calcaneal fractures (DIACFs) pose a significant therapeutic challenge. Open reduction and internal fixation (ORIF) via an extensile lateral approach (ELA) has been the historical standard, demonstrating improved functional outcomes.[1–5] However, it carries a substantial risk of wound complications, with reported rates up to 11–27%.[2, 6–9] Consequently, minimally invasive surgery (MIS) techniques, including percutaneous reduction and fixation (PRF) and the sinus tarsi approach (STA), have gained prominence for their ability to minimize soft-tissue insult, reduce infection rates, shorten hospital stay, and facilitate earlier rehabilitation.[10, 11] Consequently, the application of MIS for calcaneal fractures is increasing.

Initially, MIS was primarily indicated for simple tongue-type fractures.[12–15] As surgical experience has accumulated, its use has expanded to include selected Sanders type II and some type III fractures.[10, 16–25] Despite this, considerable debate remains, especially for complex Sanders type III fractures with a comminuted tuberosity, where achieving anatomic reduction through limited exposures is technically demanding. A clear consensus on the definitive indications for MIS versus ELA is lacking.

A fundamental principle enabling MIS is ligamentotaxis—the use of the intact surrounding soft-tissue envelope (ligaments, joint capsule, plantar aponeurosis) to translate traction forces into reduction forces on the fracture fragments.[26] The critical prerequisite for successful ligamentotaxis is a stable, sizeable fulcrum point. Anatomically, the calcaneal tuberosity serves as this essential lever. Effective single-point, two-point (with tibia or talus), or three-point traction manoeuvres all depend on an intact tuberosity fragment.[14, 16–18, 27–33] Conversely, a comminuted tuberosity fragment disrupts this mechanical linkage, rendering ligamentotaxis less reliable and often necessitating direct visualization and reduction via an open approach.

While the integrity of the tuberosity is implicitly acknowledged in the context of ligamentotaxis, its systematic evaluation as a standalone preoperative criterion has not been widely studied. We hypothesized that the integrity of the calcaneal tuberosity, as the direct enabler of ligamentotaxis, could serve as a more mechanistic and reliable preoperative criterion for selecting the surgical approach than fracture classification alone. This study aimed to evaluate the clinical and radiographic outcomes of a tuberosity integrity-based treatment algorithm compared to the conventional Sanders classification-based algorithm.

## Methods

### Study design and participants

This single-centre retrospective case–control study was conducted at a level I trauma centre and approved by the Institutional Review Board. The requirement for informed consent was waived by the same committee due to the retrospective nature of the study. We screened all patients who underwent surgical treatment for a calcaneal fracture between January 2019 and December 2022. Inclusion criteria were: (1) unilateral, closed, displaced intra-articular calcaneal fracture; (2) age ≥ 18 years; (3) time from injury to surgery ≤ 14 days; (4) availability of a preoperative computed tomography (CT) scan confirming the presence of a discernible tuberosity fragment. Exclusion criteria included: (1) polytrauma with other major fractures; (2) history of prior ipsilateral calcaneal surgery; (3) severe osteoporosis (defined as a bone mineral density T-score ≤ −3.0 at the lumbar spine or femoral neck, or radiographic evidence of multiple fragility fractures); (4) incomplete medical records.

### Definitions: calcaneal tuberosity fragment

**Fig. 1:**
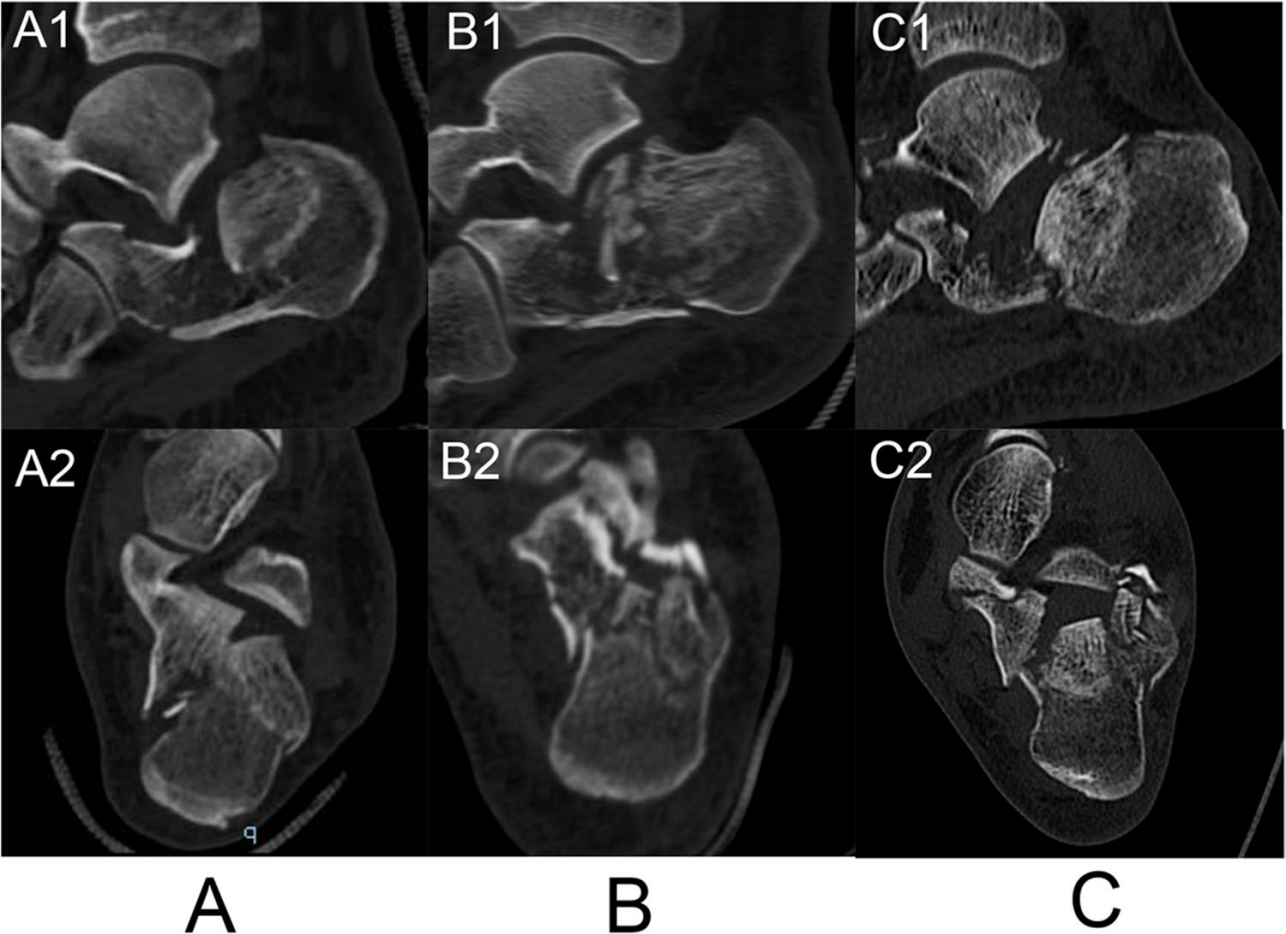
Intact tuberosity fragment in joint depression-type fractures. **A**–**C** Sanders types II, III, and IV, respectively. (A1, B1, C1) Sagittal CT scans. (A2, B2, C2) Coronal CT scans. CT, computed tomography

**Fig. 2:**
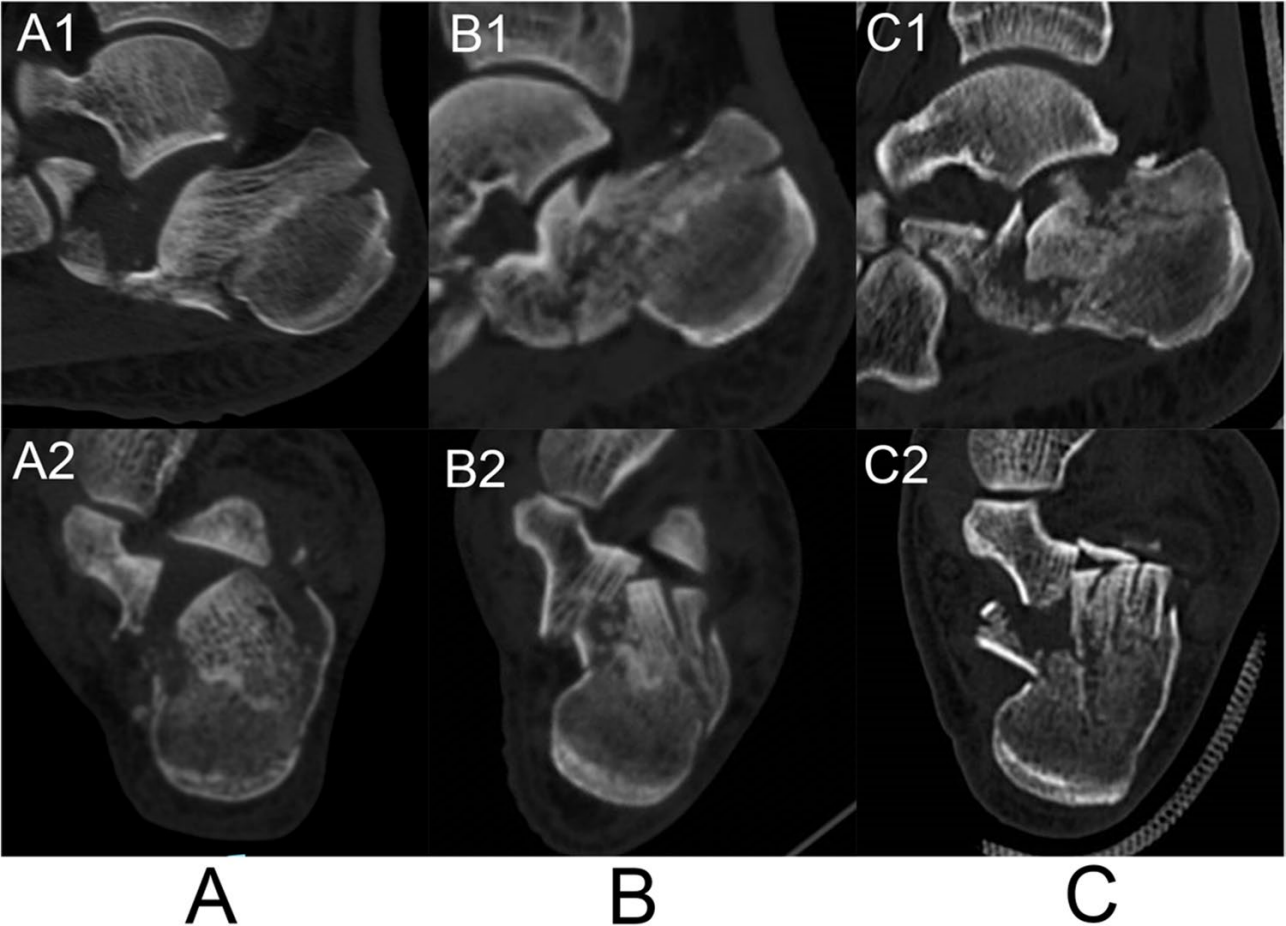
Intact tuberosity fragment in tongue-type fractures. **A**–**C** Sanders types II, III, and IV, respectively. (A1, B1, C1) Sagittal CT scans. (A2, B2, C2) Coronal CT scans. CT, computed tomography

**Fig. 3:**
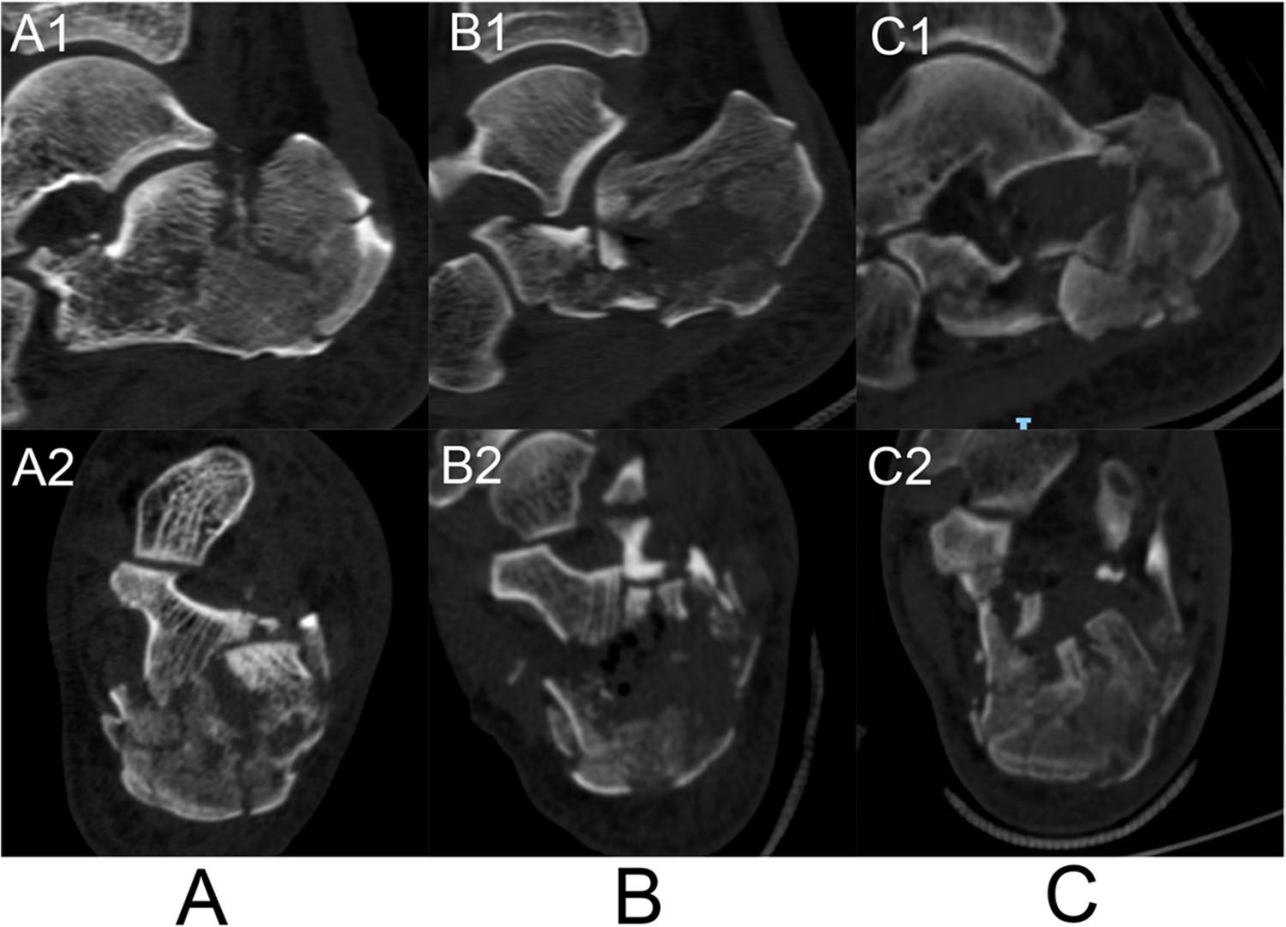
Comminuted tuberosity fragment. **A**–**C** Sanders types II, III, and IV, respectively. (A1, B1, C1) Sagittal CT scans. (A2, B2, C2) Coronal CT scans. CT, computed tomography

The calcaneal tuberosity fragment is defined as the portion of the posterolateral calcaneus—created by the primary fracture line and separated by a secondary fracture line—that lacks an articular surface (Fig. 1-3). Intact tuberosity was classified into two biomechanically competent patterns: the entire tuberosity(Fig. 1), often seen in Essex-Lopresti joint depression-type fractures, and the partial tuberosity fragment where the fracture line lies proximal to the Achilles tendon insertion (Fig. 2), typically seen in tongue-type fractures. Both configurations are considered capable of providing a stable fulcrum. Comminuted tuberosity was defined as the tuberosity being fractured into two or more discrete major fragments (Fig. 3), indicating loss of structural unity and inability to effectively transmit ligamentotaxis forces.

Tuberosity integrity was assessed on preoperative CT scans by a junior attending surgeon, with confirmation by a senior trauma professor. No formal interobserver or intraobserver reliability statistics were calculated for this specific study, which we acknowledge as a limitation. However, the assessment of tuberosity integrity was performed by consensus of two experienced surgeons, reflecting our clinical routine. We recognize that future studies should include a formal reliability analysis to enhance the reproducibility of this decision-making criterion.

### Treatment protocols and historical group allocation

During the study period, the surgical philosophy at our institution evolved based on accumulating experience with ligamentotaxis, creating a natural experiment for cohort comparison.

**Fig. 4:**
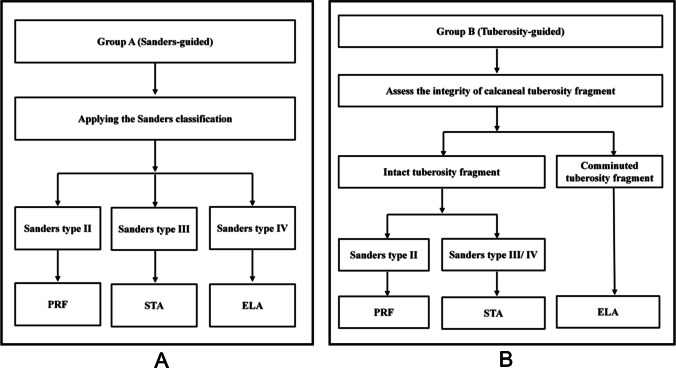
Surgical decision-making algorithms. (**A**) Algorithm for Group A (Sanders-guided). (**B**) Algorithm for Group B (tuberosity-guided)

Phase 1 (Historical Control Period: January 2019 – October 2020): The surgical approach was determined primarily by the Sanders CT classification. A standardized protocol was followed (Fig. 4A): Sanders type II and III fractures underwent MIS (PRF for type II and STA for type III), while Sanders type IV fractures underwent ORIF via an ELA. All consecutive eligible patients treated during this period constituted Group A (Sanders-guided, n = 51).

Phase 2 (New Protocol Period: November 2020 – December 2022): Based on retrospective analysis of outcomes and a refined understanding of tuberosity biomechanics, a new institutional protocol was adopted (Fig. 4B). The approach was determined by calcaneal tuberosity integrity: patients with an intact tuberosity received MIS (PRF for type II, STA for type III and Ⅳ); patients with a comminuted tuberosity (≥ 2 fragments) received an ELA, irrespective of Sanders type. All consecutive eligible patients treated under this protocol constituted Group B (Tuberosity-guided, n = 67).

Group allocation was thus determined solely by the date of surgery relative to the protocol change, minimizing selection bias related to surgeon preference or patient characteristics.

### Surgical techniques

All surgeries were performed by one of three fellowship-trained orthopaedic trauma surgeons proficient in both techniques. The choice of MIS technique was standardized based on fracture pattern and group allocation. In Group A (Sanders-guided), PRF was used for Sanders type II fractures, and STA for Sanders type III fractures. In Group B (tuberosity-guided), among patients deemed suitable for MIS (i.e., those with an intact tuberosity), PRF was used for Sanders type II fractures, while STA was used for Sanders type III and type IV fractures. Regarding fixation, MIS cases were stabilized with cannulated screws when the sustentaculum tali fragment had sufficient bone volume; otherwise, minimally invasive plates were employed. For fractures treated via ELA, anatomical plates were applied.

**Fig. 5:**
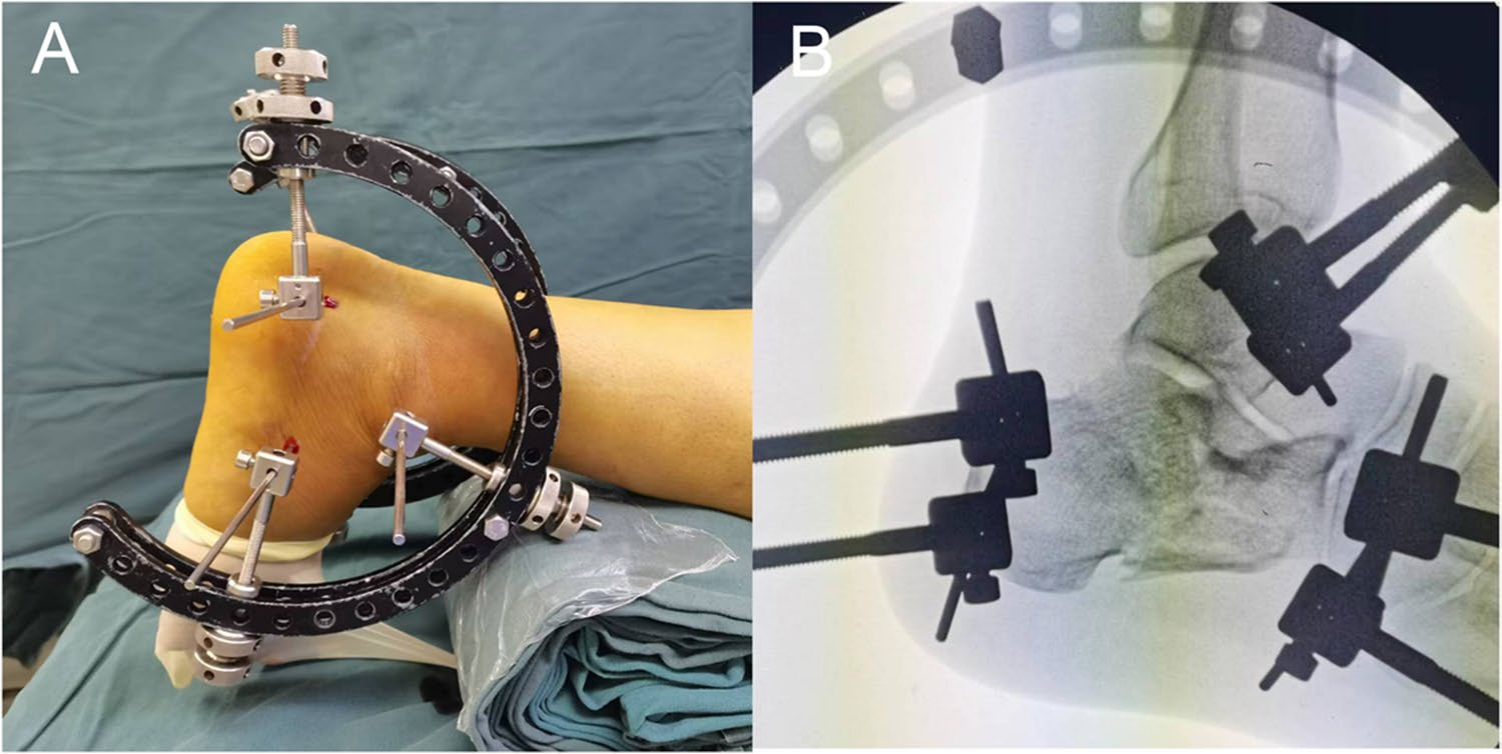
Intraoperative application of ligamentotaxis using a three-point traction technique. (**A**) Schematic illustration of distractor placement: one pin in the calcaneal tuberosity, one in the talar neck, and one in the cuboid. (**B**) Clinical photograph showing restoration of calcaneal morphology under traction

Operative Techniques: For MIS, a standardized three-step technique was employed, as previously detailed:[31, 32] (1) restoration of calcaneal morphology via ligamentotaxis using three-point traction (Fig. 5); (2) reduction of the posterior facet percutaneously or via STA, according to fracture pattern; and (3) percutaneous "tripod" screw fixation using medial longitudinal, sustentaculum, and lateral longitudinal screws. For ORIF, a standard ELA[34] was used to achieve direct visualization and anatomical reconstruction of the tuberosity and articular surface.

**Fig. 6:**
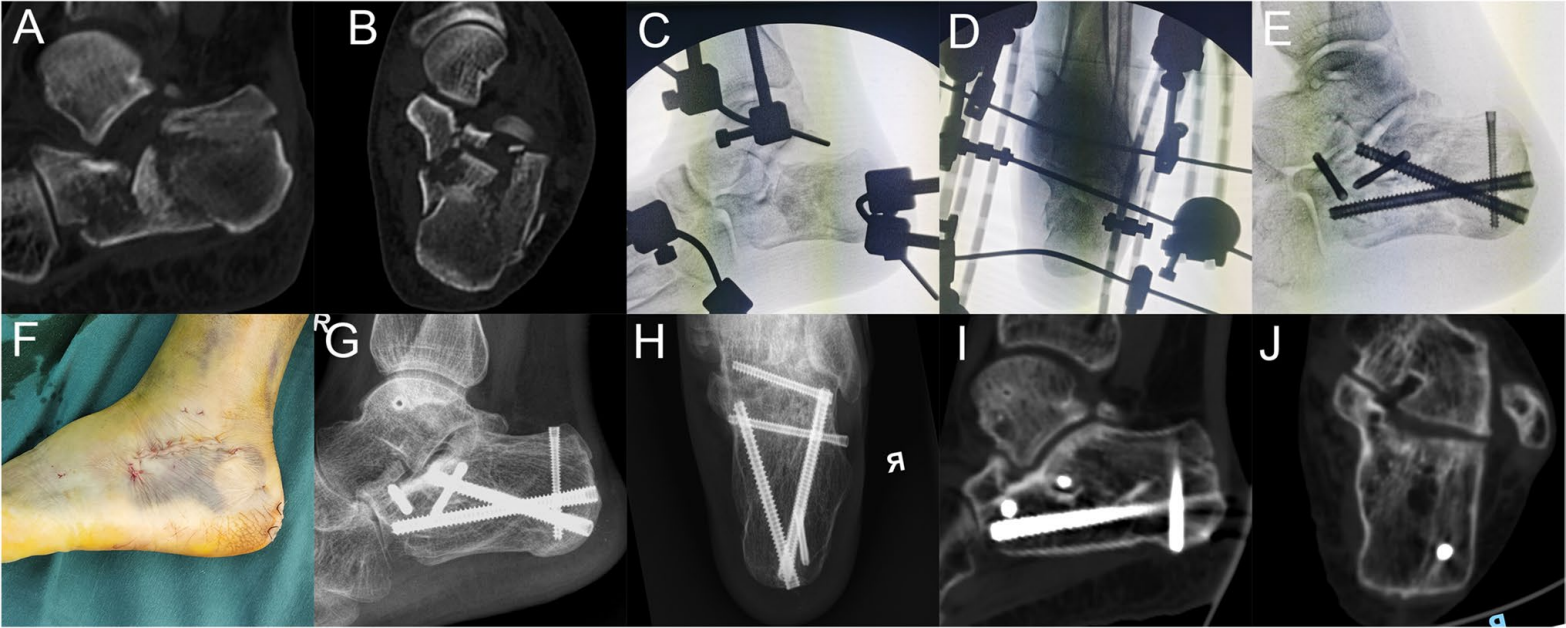
Case example of a Sanders type IV DIACF with an intact tuberosity treated with MIS. (A, B) Preoperative CT scans showing the fracture with an intact tuberosity fragment. (C–F) Intraoperative photographs showing sequential steps: restoration of calcaneal morphology via ligamentotaxis, articular reduction through STA, and percutaneous screw fixation. (G–J) Follow-up radiographs and CT scans at 27 months demonstrating maintained reduction and complete fracture healing. CT, computed tomography; DIACF, displaced intra-articular calcaneal fracture; MIS, minimally invasive surgery; STA, sinus tarsi approach

Representative cases are illustrated in Fig. 6.

### Postoperative rehabilitation and follow-up protocol

All patients adhered to a standardized, phased rehabilitation protocol. Immediate non-weight-bearing ankle and toe mobilization commenced within 24 h postoperatively to mitigate complications. Partial weight-bearing (25–50% of body weight) was initiated at six to eight weeks, progressing to full weight-bearing by ten to 12 weeks upon confirmation of radiographic union. Structured follow-up evaluations were conducted at one, three, six, 12 months, and annually thereafter. Assessments included radiographic analysis (Böhler's angle, posterior facet alignment), functional outcome scoring and surveillance for complications (wound issues, sural nerve symptomatology, post-traumatic arthritis).

### Outcome measures

Data were extracted from electronic medical records and picture archiving and communication systems.

1. Primary Outcomes: Operative time (skin incision to closure), preoperative waiting time (admission to surgery), length of hospital stay.
2. Secondary Outcomes:

Radiographic Assessment: Postoperative and final follow-up radiographs/CTs were evaluated by two blinded surgeons. Measurements included Böhler’s angle and posterior facet step-off (≤ 2 mm defined as satisfactory reduction[35, 36]).

Functional Assessment: At minimum 12-month follow-up, function was evaluated using the American Orthopaedic Foot & Ankle Society (AOFAS) Ankle-Hindfoot Score and the Maryland Foot Score (MFS). Pain was assessed using a Visual Analog Scale (VAS, 0–10) at rest and during weight-bearing.

Complications: Recorded complications within one year included wound-edge necrosis, superficial/deep infection, sural nerve injury (persistent numbness/dysaesthesia), and radiographic evidence of post-traumatic subtalar arthritis.

### Statistical analysis

Statistical analysis was performed using SPSS software (version 24.0). Continuous variables were expressed as mean ± standard deviation and compared using independent samples t-test. Categorical variables were expressed as frequencies and percentages and compared using Chi-square test. A p-value < 0.05 was considered statistically significant.

## Results

### Baseline demographics and fracture characteristics

**Fig. 7:**
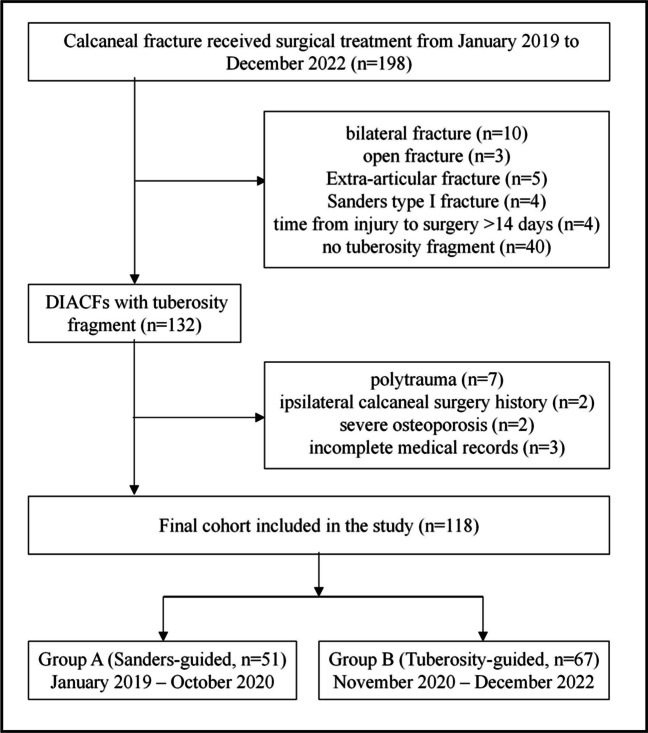
Flowchart illustrating patient screening and enrollment. DIACFs, displaced intra-articular calcaneal fractures

A total of 118 patients met the inclusion criteria and were analyzed (Fig. 7). The baseline characteristics were well-balanced between the two groups (Table 1). The mean age was 46.2 ± 12.3 years, with a male predominance (83.9%). The distribution of Sanders types (II, III, IV) and Essex-Lopresti classifications (tongue-type vs. joint depression-type) showed no statistically significant differences. The proportion of patients with a comminuted tuberosity was also similar (Group A: 5/51, 9.8%; Group B: 6/67, 9.0%; p = 0.875). Preoperative waiting time, total hospital stay, and follow-up duration were comparable.

**Table 1:** Patient demographics and perioperative data

	Group A	Group B	P value
Gender, n (M/F)	44/7	55/12	0.540^a^
Age* (yr)	46.6 ± 13.5	45.7 ± 11.3	0.686^b^
Fracture side, n (Left/Right)	23/28	29/38	0.844^a^
Diabetes	6	10	0.619^a^
Smoker	8	14	0.472^a^
Peripheral vascular disease	0	1	0.381^a^
Sanders Classification, n			
TypeⅡ	31	47	0.432^a^
Type Ⅲ	14	16	
Type Ⅳ	6	4	
Essex-Lopresti Classification, n			
Tongue-type	28	33	0.543^a^
Joint depression-type	23	34	
Preoperative Waiting Time*(days)	4.0 ± 1.9	4.0 ± 1.9	0.931^b^
Opertive Time* (min)	81.8 ± 12.4	78.9 ± 10.5	0.167^b^
Length of stay* (days)	8.6 ± 2.8	8.6 ± 2.8	0.915^b^
Follow Up* (mo)	22.4 ± 8.8	25.0 ± 10.6	0.167^b^
Approach, n			
PRF	25	37	0.769^a^
STA	20	24	
ELA	6	6	
Fixation Method, n			
CS	40	59	0.245^a^
MIP	5	2	
AP	6	6	

Abbreviations: *AP* anatomical plate, *CS* cannulated screw, *ELA* extended lateral approach, *MIP* minimally invasive plate, *PRF* percutaneous reduction and fixation, *STA* sinus tarsi approach.

*The values are given as the mean and the standard deviation; ^a^Chi-square test; ^b^Independent samples t-test.

### Surgical and perioperative outcomes

The overall mean operative time was not significantly different between Group A (81.8 ± 12.4 min) and Group B (78.9 ± 10.5 min, p = 0.167) (Table 1). The PRF/STA distribution did not differ significantly between groups (Group A: 25/45 [55.6%]; Group B: 37/61 [60.7%]; p = 0.598, Table 1). An analysis of the predefined subgroup of patients with comminuted tuberosity fractures (n = 11)—a group too small for definitive statistical inference—suggested a shorter operative time in the tuberosity-guided group (Group B, n = 6: 93.5 ± 6.1 min) compared to the Sanders-guided group (Group A, n = 5: 103.2 ± 4.7 min), with a mean difference of 9.7 min (p = 0.017) (Table 2). Given the very small subgroup size (n = 11), this finding should be interpreted as preliminary and hypothesis-generating, not as conclusive evidence of superiority.

**Table 2:** Baseline characteristics and clinical outcomes of patients with comminuted tuberosity

	Group A	Group B	P value
Sanders Classification			
TypeⅡ	0	1	0.517^a^
Type Ⅲ	2	3	
Type Ⅳ	3	2	
Essex-Lopresti Classification			
Tongue-type	3	4	0.819^a^
Joint depression-type	2	2	
Preoperative Waiting Time* (days)	7.4 ± 1.7	8.0 ± 1.4	0.535^b^
Opertive Time* (min)	103.2 ± 4.7	93.5 ± 6.1	0.017^b^
Length of stay* (days)	14.6 ± 2.1	15.3 ± 2.7	0.628^b^
Follow Up* (mo)	24.4 ± 9.3	26.0 ± 12.3	0.817^b^
BÖhler angle* (deg)			
Preoprative	6.8 ± 5.6	7.3 ± 5.1	0.873^b^
Postoperative	28.0 ± 1	25.7 ± 3.1	0.148^b^
Final Follow-up	28.2 ± 0.8	26.8 ± 2.8	0.321^b^
Satisfactory Rate of Facet Reduction			
Postoperative	60.0%	83.3%	0.387^a^
Final Follow-up	80.0%	83.3%	0.887^a^
AOFAS*	80.4 ± 10.9	82.5 ± 10.3	0.750^b^
MFS*	78.8 ± 11.1	81.0 ± 8.9	0.723^b^
VAS*			
Pain at Rest	1.2 ± 1.1	0.8 ± 1.0	0.573^b^
Pain during Weight-bearing	3.4 ± 0.9	3.0 ± 0.6	0.407^b^
Wound-healing complications	40.0%	16.7%	0.387^a^
Sural nerve injury, n(%)	20.0%	16.7%	0.887^a^
Traumatic arthritis, n(%)	20.0%	16.7%	0.887^a^

Abbreviations: *AOFAS* American Orthopaedic Foot & Ankle Society, *MFS* Maryland Foot Score, *VAS* visual analogue scale.

*The values are given as the mean and the standard deviation; ^a^Chi-square test; ^b^Independent samples t-test.

### Radiographic outcomes

Radiographic assessment demonstrated comparable quality of reduction between the two treatment algorithms (Table 3). Postoperative Böhler’s angles were satisfactorily restored in both groups (Group A: 27.2 ± 4.4°; Group B: 27.4 ± 4.8°; p = 0.808). The rate of satisfactory reduction of the posterior facet (step-off ≤ 2 mm) immediately postoperatively was 90.2% in Group A and 91.0% in Group B (p = 0.875). These results were maintained at the final follow-up, with no significant loss of reduction observed in either group.

**Table 3:** Preoperative, immediate postoperative and final follow-up radiographic measurements

	Group A	Group B	*P* value
BÖhler angle* (deg)			
Preoprative	0.7 ± 16.0	5.3 ± 13.3	0.091^b^
Postoperative	27.2 ± 4.4	27.4 ± 4.8	0.808^b^
Final Follow-up	27.3 ± 3.7	27.4 ± 4.7	0.862^b^
Satisfactory Rate of Facet Reduction			
Postoperative	90.2%	91.0%	0.875^a^
Final Follow-up	92.2%	91.0%	0.830^a^

*The values are given as the mean and the standard deviation; ^a^Chi-square test

^b^Independent samples t-test

### Functional outcomes

At a mean follow-up of 23.7 months, functional outcomes were excellent and equivalent between groups (Table 4). The mean AOFAS scores were 88.3 ± 7.7 in Group A and 88.3 ± 7.2 in Group B (p = 0.980). Similarly, the mean Maryland Foot Scores were 86.4 ± 7.9 and 86.3 ± 6.8, respectively (p = 0.919). Patient-reported pain levels, both at rest and during weight-bearing, were low and did not differ significantly.

**Table 4:** Functional outcome scores at final follow-up

	Group A	Group B	P value
AOFAS*			
Average	88.3 ± 7.7	88.3 ± 7.2	0.980^b^
Good to Excellent Rate (%)	92.2%	92.5%	0.939^a^
MFS*			
Average	86.4 ± 7.9	86.3 ± 6.8	0.919^b^
Good to Excellent Rate (%)	90.2%	92.5%	0.651^a^
VAS*			
Pain at Rest	0.6 ± 0.8	0.6 ± 0.7	0.826^b^
Pain during Weight-bearing	2.3 ± 1.3	2.3 ± 1.3	0.774^b^

Abbreviations: *AOFAS* American Orthopaedic Foot & Ankle Society, *MFS* Maryland Foot Score, *VAS* visual analogue scale.

*The values are given as the mean and the standard deviation; ^a^Chi-square test; ^b^Independent samples t-test.

### Complications

The overall complication profile was favourable and similar between groups (Table 5). The total wound complication rate (including necrosis and superficial infection) was 9.8% in Group A and 6.0% in Group B (p = 0.437). There were no deep infections. Sural nerve injury occurred in 5.9% of patients in Group A and 3.0% in Group B (p = 0.648). The incidence of post-traumatic arthritis was low and statistically similar across groups (2.0% VS 1.5%, p = 0.845).

**Table 5:** Complications

	Group A	Group B	P value
Wound-healing complications, n(%)			
Wound-edge necrosis	4 (7.8%)	3 (4.5%)	0.443^a^
Superficial infection	1 (2.0%)	1 (1.5%)	0.845^a^
Deep infection	0.0%	0.0%	NA
All	5 (9.8%)	4 (6.0%)	0.437^a^
Sural nerve injury, n(%)	2 (5.9%)	2 (3.0%)	0.439^a^
Traumatic arthritis, n(%)	1 (2.0%)	1 (1.5%)	0.845^a^

^a^Chi-square test; ^b^Independent samples t-test

### Subanalysis of wound complications and neurological outcomes by approach and tuberosity integrity

In the entire cohort of 118 patients, a detailed subanalysis revealed significant findings regarding complications relative to surgical approach and tuberosity status. This subanalysis, based on a limited number of patients with comminuted tuberosity (n = 11), must be interpreted with caution. It indicated a significantly higher wound complication rate when STA was used for such fractures (2/2, 100%) compared to ELA (1/9, 11.1%; p = 0.011) (Supplemental Table 1). Furthermore, examining all 44 patients who underwent STA, wound complications occurred in both patients with a comminuted tuberosity (2/2, 100%) but in only four of the 42 patients with an intact tuberosity (4/42, 9.5%; one superficial infection and three edge necrosis), a difference that was statistically significant (p < 0.001) (Supplemental Table 2).

An analysis of sural nerve injury within the STA subgroup also demonstrated a clinically notable trend. The incidence of nerve injury was substantially higher in patients with a comminuted tuberosity (1/2, 50%) compared to those with an intact tuberosity (2/42, 4.8%) (Supplemental Table 2). Although this difference did not reach conventional statistical significance (p = 0.055), it suggests a potential association between tuberosity comminution and increased risk of neurological compromise when the STA is employed.

## Discussion

The principal findings of this study demonstrate that a surgical decision-making algorithm based on the preoperative integrity of the calcaneal tuberosity produces radiographic and functional outcomes that are comparable to those achieved using the well-established Sanders classification. This validates our hypothesis that tuberosity integrity is a clinically relevant and reliable preoperative indicator. Beyond achieving non-inferior outcomes, our analysis generates the hypothesis that a tuberosity-guided approach might offer practical advantages: in the surgically challenging subgroup of fractures with a comminuted tuberosity, the algorithm was associated with a trend toward reduced operative time and was correlated with a different profile of approach-related complications. However, these latter findings are based on a very small number of cases (n = 11 for comminuted tuberosity fractures) and must be viewed as exploratory.       

The reduction in operative time for fractures with a comminuted tuberosity is a pertinent preliminary finding. Prolonged surgical time is an independent risk factor for surgical site infection, increased soft-tissue trauma, and higher anaesthetic burden.[37, 38] The time saving observed in our study likely reflects the algorithm's inherent efficiency. In the Sanders-guided protocol, a surgeon encountering Sanders type Ⅱ and III fracture might proceed with an initial MIS attempt.[20–24] However, when the tuberosity is comminuted, achieving a stable reduction via MIS becomes technically demanding. This complexity not only poses a risk for extended operative time but may also increase the vulnerability to wound complications, as suggested by our subanalysis. The tuberosity-guided protocol addresses this by facilitating a definitive preoperative plan. Preoperative CT identification of tuberosity comminution directly indicates the use of an ELA, thereby circumventing the potential inefficiencies and heightened risks associated with attempting MIS in this specific setting, contributing to a more streamlined and potentially safer operative course.

Our findings thereby refine the rationale for surgical approach selection by highlighting the complementary role of tuberosity integrity to the Sanders classification. The traditional algorithm primarily uses fracture morphology to recommend MIS for Sanders type II/III and ELA for type IV.[5, 18, 20, 22, 25, 35] Our experience suggests that this can be optimized. For Sanders type II or III fractures with a comminuted tuberosity, an ELA may offer a more efficient and reliable alternative, as it avoids the technical constraints of MIS in the absence of a stable fulcrum. Conversely, for Sanders type IV fractures, MIS (e.g., STA) remains a viable and advantageous option if the tuberosity fragment is intact, as ligamentotaxis can effectively restore calcaneal morphology prior to articular reduction. Therefore, integrating tuberosity integrity into the decision-making process provides a biomechanically grounded supplement that enhances the precision of approach selection across different Sanders types.

Regarding neurological outcomes, among patients with comminuted tuberosity fractures, the incidence of sural nerve injury did not differ statistically between STA and ELA. However, a trend toward a higher rate was observed in the STA subgroup. This tendency may reflect the technical challenges inherent in managing a fragmented tuberosity through a limited exposure, which can necessitate more aggressive soft-tissue retraction or posterior extension of the incision to achieve adequate fragment control. It is critical to note that the small cohort of comminuted fractures limits definitive statistical conclusions. Nevertheless, the observed pattern, coupled with the significantly higher wound complication rate for STA in this subgroup, collectively suggests that STA may carry an increased risk of local soft-tissue complications in the setting of tuberosity comminution. These findings further support the utility of preoperative tuberosity assessment in guiding surgical approach selection, which in these cases favours ELA.

The biomechanical foundation for using tuberosity integrity is robust and directly links preoperative imaging to intraoperative technique. Ligamentotaxis is a force-coupling mechanism entirely dependent on a stable, intact tuberosity fragment to act as a fulcrum.[26] Our results confirm that when this prerequisite is met, MIS can be successfully employed across various Sanders types, achieving anatomical reduction. In contrast, when the tuberosity is comminuted, the mechanical link is broken, making indirect reduction unpredictable and justifying the need for direct visualization and reduction afforded by an ELA. This provides a clear, binary, and objective criterion that is easily assessed on routine preoperative CT scans.

The comparable functional and radiographic outcomes between the two groups underscore that the Sanders classification, while excellent for describing fracture patterns and prognosis, may not be the optimal sole criterion for selecting a surgical technique. The tuberosity-based algorithm effectively "re-categories" fractures based on a fundamental biomechanical property (fulcrum stability) rather than a purely morphological pattern. Importantly, this re-categorization did not lead to worse outcomes. On the contrary, it contributed to more efficient surgery by appropriately directing Sanders II/III fractures with comminuted tuberosity to an ELA—a decision supported by temporal efficiency and a potentially safer approach selection, though the latter requires further validation. 

## Limitations

This study has several limitations. First, its retrospective, single-centre design introduces potential selection and information bias. Crucially, the two treatment groups were derived from consecutive time periods (historical control vs. new protocol), which introduces potential temporal bias. Improvements in surgical technique or surgeon experience over the study period could have contributed to the outcomes in Group B, independent of the algorithm change. Thus, this historical cohort design limits our ability to attribute all observed differences solely to the tuberosity-guided protocol. Second, the most important statistical limitation is the very small sample size of the comminuted tuberosity subgroup (n = 11). Consequently, all subgroup findings—including the observed differences in operative time and complication rates—lack sufficient statistical power and are at high risk of type II error. They must be interpreted solely as preliminary, hypothesis-generating observations that require confirmation in adequately powered, prospective, multi-center cohorts. Third, we did not perform formal interobserver or intraobserver reliability testing for the assessment of tuberosity integrity. While the parameter was evaluated by consensus of two experienced surgeons reflecting clinical routine, the lack of quantitative reproducibility data limits the generalizability of this decision-making criterion. Future studies should include such an analysis. Finally, the mean follow-up of 23.7 months may not capture very long-term sequelae such as progressive subtalar arthritis or late hardware-related complications.

## Conclusion

In conclusion, preoperative assessment of calcaneal tuberosity integrity provides a biomechanically sound and practical adjunct to the Sanders classification for surgical approach selection in DIACFs. The proposed algorithm, which integrates this parameter, yields radiographic and functional outcomes comparable to a classification-guided approach. Incorporating tuberosity integrity into preoperative planning may offer a reliable supplement to conventional classification systems for optimizing surgical strategy. Given the preliminary nature of our subgroup findings, particularly regarding complications and operative time in comminuted tuberosity fractures, a prospective, randomized controlled trial with a pre-planned analysis of this specific fracture pattern is warranted before recommending widespread adoption.

## Supplementary Information

Below is the link to the electronic supplementary material.

ESM 1 (DOCX 12.4 KB)

## Data Availability

The data that support the findings of this study are available from the corresponding author upon reasonable request, subject to ethical/legal restrictions.

## Abbreviations

AOFAS: American Orthopaedic Foot & Ankle Society
AP: Anatomical plate
CS: Cannulated screw
CT: Computed tomography
DIACFs: Displaced intra-articular calcaneal fractures
ELA: Extended lateral approach
MFS: Maryland foot score
MIP: Minimally invasive plate
MIS: Minimally invasive surgery
ORIF: Open reduction and internal fixation
PRF: Percutaneous reduction and fixation
STA: Sinus tarsi approach
VAS: Visual analogue scale
