# Supplementary material for: The guiding significance of calcaneal tuberosity integrity in the surgical treatment of calcaneal fractures: a retrospective case–control study
Source: Int Orthop. 2026 Jun 23;50(7):1787–97. doi: 10.1007/s00264-026-06927-8 (PMC13407454; doi:10.1007/s00264-026-06927-8)
Supplement: Supplementary file 1 — (DOCX 12.4 KB) [file 264_2026_6927_MOESM1_ESM.docx]

Supplemental Table 1. Complication Profiles in Patients with Comminuted Tuberosity

|  | STA | ELA | P value |
| --- | --- | --- | --- |
| Wound-healing complications, n(%) | 2 (100%) | 1 (11.1%) | 0.011^a^ |
| Sural nerve injury, n(%) | 1 (50.0%) | 1 (11.1%) | 0.197^a^ |

Abbreviations: ELA, extended lateral approach; STA, sinus tarsi approach.

^a^Chi-square test.

Supplemental Table 2. Complication Profile Associated with the Sinus Tarsi Approach

|  | Intact tuberosity | Comminuted tuberosity | P value |
| --- | --- | --- | --- |
| Wound-healing complications, n(%) | 4 (9.5%) | 2 (100%) | 0.000^a^ |
| Sural nerve injury, n(%) | 2 (4.8%) | 1 (50.0%) | 0.055^a^ |

^a^Chi-square test.
